# Streamlining Care in Crisis: Rapid Creation and Implementation of a Digital Support Tool for COVID-19

**DOI:** 10.5811/westjem.2020.7.48537

**Published:** 2020-08-17

**Authors:** Nicholas Stark, Michaela Kerrissey, Madeline Grade, Beth Berrean, Christopher Peabody

**Affiliations:** * University of California San Francisco, Department of Emergency Medicine, San Francisco, California; † Harvard School of Public Health, Department of Health Policy and Management, Boston, Massachusetts; ‡ University of California San Francisco, School of Medicine Tech Division, San Francisco, California

## Abstract

The unprecedented COVID-19 pandemic has resulted in rapidly evolving best practices for transmission reduction, diagnosis, and treatment. A regular influx of new information has upended traditionally static hospital protocols, adding additional stress and potential for error to an already overextended system. To help equip frontline emergency clinicians with up-to-date protocols throughout the evolving COVID-19 crisis, our team set out to create a dynamic digital tool that centralized and standardized resources from a broad range of platforms across our hospital. Using a design thinking approach, we rapidly built, tested, and deployed a solution using simple, out-of-the-box web technology that enables clinicians to access the specific information they seek within moments. This platform has been rapidly adopted throughout the emergency department, with up to 70% of clinicians using the digital tool on any given shift and 78.6% of users reporting that they “agree” or “strongly agree” that the platform has affected their management of COVID-19 patients. The tool has also proven easily adaptable, with multiple protocols being updated nearly 20 times over two months without issue. This paper describes our development process, challenges, and results to enable other institutions to replicate this process to ensure consistent, high-quality care for patients as the COVID-19 pandemic continues its unpredictable course.

## INTRODUCTION

The unprecedented COVID-19 pandemic has resulted in rapidly evolving best practices for transmission reduction, diagnosis, and treatment.^1^ This has challenged emergency departments (ED) to shift from using relatively static clinical protocols to an immensely accelerated pace of creating, updating, and disseminating protocols – with daily or weekly changes for everything from personal protective equipment (PPE) to testing guidelines.

Such a challenge is not to be underestimated. Over the past two decades, many EDs have developed capacity to create well-defined protocols and train clinicians to use them, offering significant advantages in care quality.^2^–^4^ However, because protocols are typically intended to serve as fixed guidelines, they are rarely updated and require little ongoing access by clinicians after initial training. This often leads to an array of platforms housing these hospital- and ED-specific protocols, which was the case at our hospital, Zuckerberg San Francisco General (ZSFG).

The COVID-19 pandemic upended this stability and left our hospital, like many others, scrambling to adjust. With up to 30 COVID-positive patients in our hospital on a given day – and several of those in the intensive care unit on ventilators – rapidly evolving protocols made it difficult for clinicians to stay up to date with new guidelines, adding additional stress and potential for error to an already overextended system.^5^–^7^

This instability, hallmarked by daily emails and online folders overflowing with lengthy PDFs providing new guidance, inspired our team to create a solution that could equip frontline clinicians with accessible, up-to-date clinical protocols throughout the evolving COVID-19 crisis. Ultimately, we were able to create a digital support tool that centralized, digitized, and standardized resources from a broad range of print and digital platforms across our hospital through using accessible, off-the-shelf technology: zsfgCOVID, which is available online at https://zsfgCOVID.ucsf.edu. In what follows, we describe how we rapidly developed and deployed this digital tool, explore its utilization in our ED, and highlight lessons as well as a step-by-step process for teams endeavoring to develop a similar approach to maximize patient care during the next phase of the COVID-19 pandemic.

## BUILDING A COVID-19 DIGITAL SUPPORT TOOL

We used an accelerated, two-pronged approach toward building a solution: 1) engaging leadership to ensure high-level support; and (2) assembling a team to iteratively build, test, and deploy a solution using the best practices of design thinking.

### Engaging Leadership

We first approached ED leadership with our project idea, and it was received with strong support. We chose to focus on ED-specific protocols first to enable our team to quickly create, test, and implement the digital support tool in a smaller setting before expanding hospital-wide.

### Assembling a Multi-Disciplinary Team

We assembled a team of three University of California, San Francisco (UCSF) emergency physicians who work clinically at ZSFG, as well as four members of a digital product studio at the UCSF School of Medicine. Many medical centers and health systems have similar studios; internal information technology departments can also serve as a partner group.

The physicians provided the clinical perspective necessary to organize the flow of the digital support tool, while the digital product team managed the project and created the web platform. While we had a heavily resourced team, the final platform required fewer resources than we used and could easily be replicated by less resourced teams, as illustrated in Table 1.

### Using Design Thinking to Rapidly Develop a Solution

**Figure 1:**
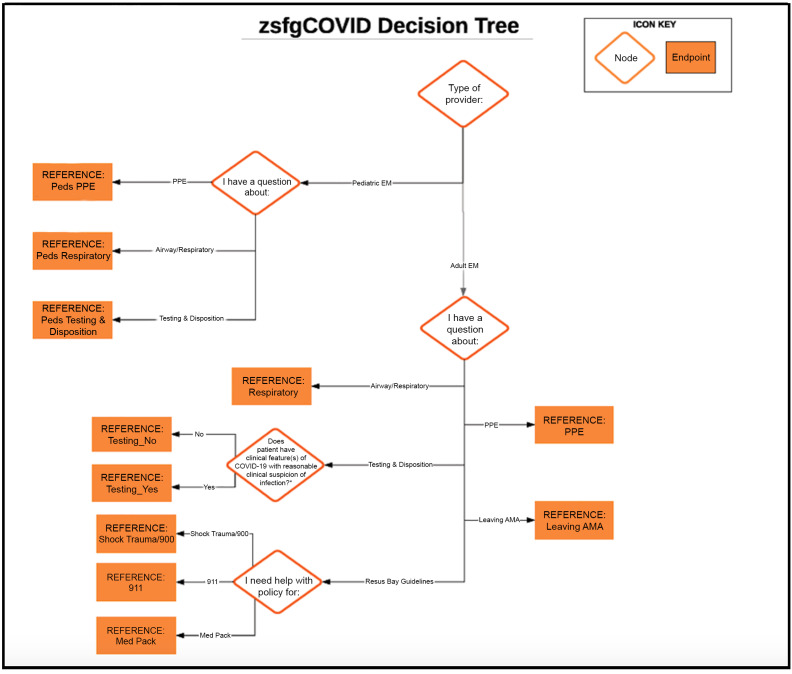
The decision tree structure to organize COVID-19 protocols. *COVID*, coronavirus 19; *PPE*, personal protective equipment; *Adult EM*, adult emergency medicine; *AMA*, against medical advice.

During our initial virtual meetings, which occurred 2–3 times per week, we used a human-centered design thinking approach to further define our problem and ideate potential solutions.^8^ After determining that a streamlined, responsive, web-based solution would likely work best, we set out to create and test a prototype. First, our team worked to build a multilevel decision tree to organize our hospital’s COVID-related protocols in a way that could eventually be translated into a digital tool (Figure 1).

**Figure 2:**
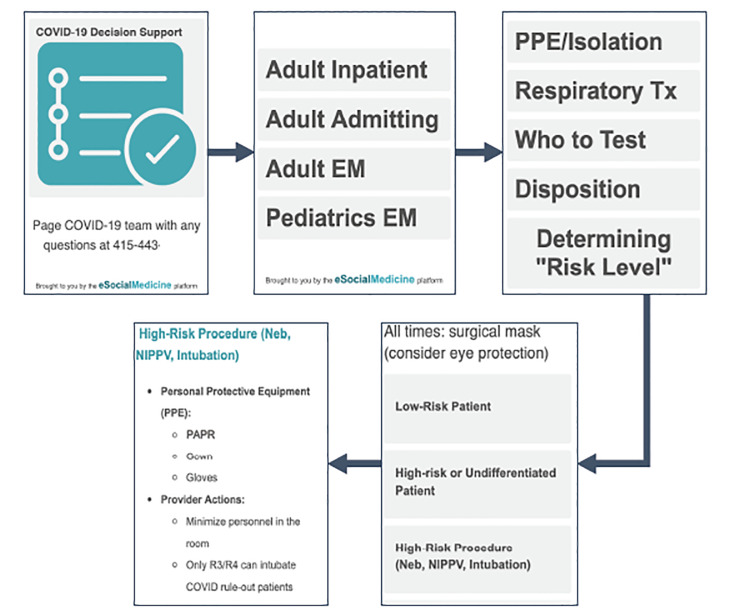
Version 1 of the digital tool. Obtaining information on which personal protective equipment to wear while intubating a patient at high risk for COVID-19 required a total of four “clicks” to reach an endpoint. *COVID-19*, coronavirus 19; *EM*, Emergency Medicine, *PPE*, personal protective equipment; *PAPR*, powered air purified respirator; *Neb*, nebulizer; *NIPPV*, non-invasive positive pressure ventilation; *Tx*, treatment.

Upon completing the first draft of the decision tree, our team split the project to work in parallel: the physicians worked to build specific endpoints for each protocol outcome, while the digital product team began to build the digital support tool. Ten days later, we had a prototype ready for testing (Figure 2).

### User Testing and Adjustments

Following five days of user testing with a group of eight resident and four attending emergency physicians, brief interviews were conducted for the purpose of rapidly collecting user feedback. The interviews, conducted by one of the authors over a five-day period, followed a semi-structured protocol; themes were recorded in memos by the lead interviewer immediately following the interviews. These interviews revealed two important insights: 1) the complex, multistep logic led to an unacceptable number of “clicks” to reach an endpoint; and 2) most users preferred broad overviews of protocols, rather than being directed to fine-tuned endpoints. From a platform maintenance perspective, the digital product team expressed concern that the multistep logic on the backend of the tool required extensive rebuilds each time the protocols changed. With some protocols changing as many as 10 times in two weeks, these technical challenges and user feedback led us back to design thinking to reframe the problem.

**Figure 3:**
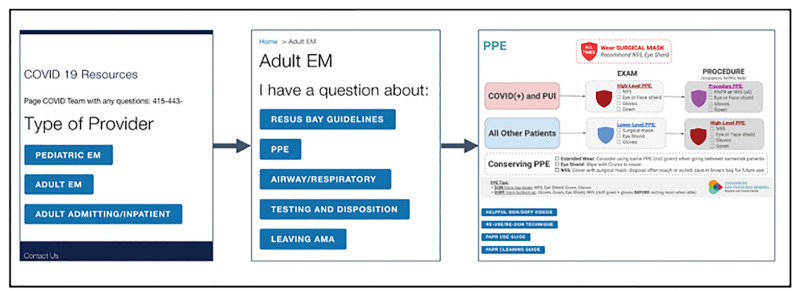
Current version of the digital tool. Obtaining information on which personal protective equipment to wear requires a total of two “clicks” to reach an endpoint. *COVID-19*, coronavirus 19; *EM*, Emergency Medicine; *PPE*, personal protective equipment; *AMA*, against medical advice; *PUI*, patient under investigation.

A virtual brainstorming meeting the following day led us to a solution: simplify the decision-support tool by creating broad, intuitive flowcharts for the hospital protocols rather than specific informational endpoints. This approach would reduce the number of “clicks” required to reach an endpoint, allow users to see broad overviews of protocols, and minimize platform rebuilds as protocols changed. Rather than lead to specific informational endpoints – such as which PPE to wear when intubating a high-risk COVID-19 patient – these new flowchart-style endpoints provide single-page overviews, such as which PPE to wear in multiple clinical scenarios (Figure 3).

**Figure 4:**
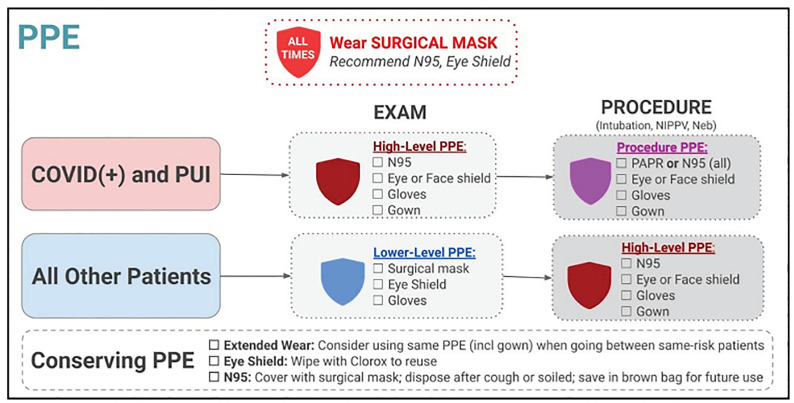
The PPE protocol flowchart in the current version of the digital tool. All aspects of the PPE protocol are displayed in a single page. *PPE*, personal protective equipment; *COVID*, coronavirus 19; *PUI*, patient under investigation.

After constructing each protocol flowchart in PowerPoint, our team uploaded the flowchart endpoints to the web platform (Figure 4).

Following these changes to the digital support tool, we approached the same 12 resident and attending emergency physicians for a second round of user testing, which revealed a dramatic improvement in perceived usability. Our team decided to move forward with an ED-wide launch on April 7, 2020; this was 26 days after initial project brainstorming began. The tool, zsfgCOVID, is named after our hospital and publicly accessible at https://zsfgCOVID.ucsf.edu.

## PRODUCT ADOPTION AND UTILIZATION

After usng a broad range of tactics to advertise the digital tool – including product demonstrations during department meetings, link-access within the electronic health record (EHR) system, flyers, and targeted email notifications– initial website usage data and user reviews indicate substantial uptake. Throughout the initial six weeks from launch, zsfgCOVID experienced 8–20 unique daily users for the 28 emergency clinicians working each day, or approximately 29–70% of daily clinicians.

Our team also conducted an institutional review board-exempt survey among emergency physicians to assess perceptions of the platform. The survey was created by adapting previously developed and validated survey measures where possible, particularly for more subjective measures such as perceived usefulness.^9^ After cognitive testing with two residents and one attending physician over a two-day period, which resulted in minimal updates to the survey measures for clarity in language, the online Qualtrics (Provo, UT) survey was emailed to 90 resident and attending physicians who work clinically in the ZSFG ED (Appendix A). The survey, accessible for one week with two email reminders, garnered a total of 28 responses for a response rate of 31.1%.

Of the physicians surveyed, 57.4% reported lacking confidence in accessing up-to-date COVID workflows and policies prior to implementation of the digital tool; 100% responded “agree” (32.1%) or “strongly agree” (67.9%) that the digital tool has made it easier to access up-to-date COVID-related protocols, and 100% “agree” (50.0%) or “strongly agree” (50.0%) that the platform was useful in their job. In addition, 78.6% responded “agree” (35.7%) or “strongly agree” (42.9%) that the platform has affected their management of patients with COVID-19 infections. For example, one clinician commented that the platform “really helped with my ability to safely discharge a homeless patient to an isolation shelter.” Other users have noted that the platform has “helped me determine who I should be testing for COVID-19, and which type of test I should order,” as well as “kept me up-to-date on which PPE I should be wearing in different clinical scenarios.”

Clinicians reported using the digital tool often, with 85.7% using the platform at least once per week. The platform has a net promoter score of 71%, which falls in the “Excellent” category. User feedback indicates that, in the future, clinicians would like to see additional functionality added to the digital tool, such as a way to link protocols to current scientific evidence. Our team plans to work to incorporate this feedback as the COVID-19 pandemic evolves.

## LESSONS LEARNED

Our team learned several lessons throughout the development and deployment process, which may aid other institutions as they work to develop similar digital support tools. These lessons include the following:

- **Engaging appropriate stakeholders during a hectic time.** Our team first reached out to ED leadership with whom we had relationships. These ED leaders became key, invested stakeholders who were able to connect us with other hospital leaders to broaden support for the digital support tool.
- **Developing a user-friendly, clinician-focused platform.** Initial user testing demonstrated that the first version of the digital support tool required too many “clicks” to reach an endpoint. The development of a simpler, more user-friendly final product occurred through multiple iterations based on user feedback from targeted interviews.
- **Using a straightforward web content management platform.** Our team initially built a heavily logic-based web platform foundation in the Qualtrics survey system.^10^ However, this approach quickly became unsustainable due to the extensive rebuilds required each time a protocol changed. Ultimately, our team found that a common web content management platform, such as Drupal (Antwerp, Belgium) was easier to use and maintain.^11^
- **Ensuring accurate, daily updates to changing protocols.** To ensure all protocols are up to date, we rely heavily on the physicians on our team. Through close communication with hospital leadership, the physicians make updates to the protocol flowcharts as recommendations evolve. Both the physicians and digital product studio members have been trained in uploading the updated protocol flowcharts to the web platform; this flattened organizational structure has enabled rapid turnarounds each time recommendations change.
- **Earning clinician trust for a new digital tool.** Top-down support was key for earning clinicians’ trust and encouraging them to use the platform. Maintaining close relationships with hospital leadership and ensuring accurate information on the digital support tool is vital.
- **Spreading the word in an information-saturated landscape.** Our team quickly realized that purely email dissemination of the digital support tool would likely lead to underutilization or failure. By thinking beyond traditional information dissemination tactics, we were able to give live demonstrations of the platform at several department-wide meetings, integrate a link to the tool in our EHR system, and post flyers throughout our ED. These tactics, combined with targeted email reminders to clinicians working on a given week, have led to high utilization.

### Application to Other Institutions

As the COVID-19 pandemic continues its unpredictable course over the coming months to years, a centralized information source that equips clinicians with up-to-date information for the care of COVID-19 patients can help improve patient care. We believe that our approach to building a centralized, digitized, and standardized resource platform through using off-the-shelf technology is applicable across academic and community settings. We recommend the following steps for institutions interested in building a similar digital support tool:

1. Talk with frontline clinicians to determine whether similar challenges with protocol management are present at your hospital.
2. Identify and engage motivated team members, including at least one clinician and one member versed in basic website design.
3. Start small. Consider beginning with a single unit or service, rather than attempting hospital-wide implementation from the start.
4. Engage leadership at the unit or service level first, and augment to hospital-level leadership with unit leadership’s support. Leadership buy-in is key for long-term success.
5. Consolidate the existing COVID-related protocols at your hospital, and develop a decision tree to outline where each protocol should be housed on the web platform.
6. Standardize the protocols into easy-to-use flowcharts. Our team used PowerPoint for this process.
7. Build a web-based platform to house the protocols. The organization of the platform will be based on the decision tree you develop. Our team used Drupal for this step.^11^
8. Launch the initial version of your digital tool quickly, and test with a small group of clinicians. Briefly interview these clinicians after they use the tool to gain insight into areas for improvement.
9. Adjust the digital tool as needed based on user feedback.
10. Launch the digital tool for a broader audience, in coordination with hospital leadership to ensure support.

## LIMITATIONS

While our team was able to rapidly develop a novel digital support tool to aid our hospital’s response to the COVID-19 pandemic, our user testing and surveying processes exhibit several limitations. First, our initial semi-structured interviews were targeted to updating the digital tool rather than more deeply exploring how respondents felt about and experienced the platform. Second, with a relatively low survey-response rate, our survey results may be subject to nonresponse bias as clinicians who have not used or do not like the platform may have been less likely to respond to the survey. Finally, although most survey respondents reported that the platform affected their management of patients with COVID-19 infections, our team did not assess outcome measures such as differences in PPE use or disposition times for clinicians who use the platform. Future research can explore the more complex relationships between these evolving digital tools and clinicians’ experience of – and effects on – patient care.

## CONCLUSION

The COVID-19 pandemic will continue to affect patients and hospital systems for the foreseeable future, and it is important for clinicians to have easy access to up-to-date hospital protocols to provide exceptional patient care. Our team’s experience has shown that simple, out-of-the-box web technology can serve as a conduit to transform typically static hospital protocols into rapidly-evolving guidelines that clinicians can access within moments. We are hopeful that, through developing similar digital support tools, other institutions are able to provide similar support to frontline clinicians throughout the COVID-19 era.

## Supplementary Information



## Figures and Tables

**Table 1:** Example team roles, time commitments, and costs to develop a digital tool for patient care.

Title	Role Description	Total Hours	Estimated Cost
Project Manager	Oversee project timeline, coordinate meetings, monitor progress, supervise budget, manage platform revisions.	25–35	$40–50/hour
Platform Developer	Build web-based platform, assist with protocol format/design, maintain platform as needed.	30–40	$75–100/hour
Clinician	Consolidate COVID-19 protocols, organize protocol flow, standardize protocol format, update protocols as needed.	30–40	$100–200/hour
