# Supplementary material for: Streamlining Care in Crisis: Rapid Creation and Implementation of a Digital Support Tool for COVID-19
Source: West J Emerg Med. 2020 Aug 17;21(5):1095–101. doi: 10.5811/westjem.2020.7.48537 (PMC7514410; doi:10.5811/westjem.2020.7.48537)
Supplement: Supplementary file 1 [file wjem-21-1095-s001.pdf]

APPENDIX A: zsfgCOVID Decision Support Tool User Survey

1) **Demographic Information**
   a. Role
   b. Residency Graduation Year (if applicable)

2) **Prior to the *zsfgCOVID platform,* I felt confident in accessing up-to-date COVID-related workflows and policies at ZSFG.**
   Strongly Disagree   Disagree   Agree   Strongly Agree

3) **Since the launch of the *zsfgCOVID* platform, I am able to more easily access up-to-date COVID-related workflows and policies at ZSFG.**
   Strongly Disagree   Disagree   Agree   Strongly Agree

4) **I find the *zsfgCOVID* platform useful in my job.**
   Strongly Disagree   Disagree   Agree   Strongly Agree

5) **The *zsfgCOVID* platform has affected my management of COVID patients.**
   Strongly Disagree   Disagree   Agree   Strongly Agree

6) **I use the *zsfgCOVID* platform approximately:**
   Never   Once per month   Once per week   Multiple times per week   Daily

7) **Select the components of the *zsfgCOVID* platform you find most useful (up to 3).**
   Equipment-related protocols / Testing-related protocols / Treatment-related protocols / Quick-links

8) **How likely is it that you would recommend the *zsfgCOVID* platform to a colleague?**
   Very Unlikely   Neutral   Very Likely
   0   1   2   3   4   5   6   7   8   9   10

9) **What would you like to see improved or added to the *zsfgCOVID* platform?** (short answer)
